# Sex for seniors: how physicians discuss older adult’s sexuality

**DOI:** 10.1186/s13584-020-00366-5

**Published:** 2020-02-21

**Authors:** Ateret Gewirtz-Meydan, Inbar Levkovich, Moshe Mock, Uri Gur, Khaled Karkabi, Liat Ayalon

**Affiliations:** 1 Sex and Couples Therapy Unit, Meir Medical Center, Kfar Saba, Israel; 2 Oranim Academic College of Education, Haifa, Israel; 3 Oncosexology Unit, Sanz Medical Center, Laniado Hospital, Netanya, Israel; 4 Urology Department, Meir Medical Center, Kfar Saba, Israel; 5 Department of Family Medicine, The Ruth & Bruce Rappaport Faculty of Medicine, The Technion-Israel Institute of Technology, Clalit Health Services, Western Galilee District, Haifa, Israel; 6 Louis and Gabi Weisfeld School of Social Work, Bar Ilan University, Ramat Gan, Israel

**Keywords:** Physicians, Older adults, Sexual function, Biomedicalization, Qualitative

## Abstract

**Background:**

This study examined physicians’ perspectives on sexuality in later life.

**Methods:**

In-depth interviews were conducted among 38 physicians with various specialties and they were asked to discuss sexuality in later life within the medical context.

**Results:**

Perceptions on older adult’s sexuality emerged from the interviews were organized into three themes: What, why and how. *What*, referred to physician’s definition to what role sexuality plays in later life and what is considered *sex*. *Why*, referred to the reasons why physicians assumed older adults experience sexual difficulties, and how these assumptions effect the diagnostic process. *How,* referred to how sexual difficulties were treated by physicians. Physicians employed a bio-medical approach when treating older, as compared to young adults with sexual dysfunction.

**Conclusions:**

The findings highlight a potential for differential treatment of older adults, based on age, rather than on other objective reasons.

## Introduction

Research has demonstrated that negative attitudes towards later life sexuality still exist within the medical profession [7, 23]. A qualitative study conducted among general practitioners [14] revealed ageist attitudes toward sexuality among older adults. The discussion of sexual health issues was perceived as more relevant to younger patients than to older patients. Moreover, sex was not recognized as an appropriate topic of discussion with older adults. In practice, the attitudes of professionals can have a powerful impact on diagnosis and treatment processes. A study conducted in the UK revealed age bias among psychiatrists, who were more likely to take sexual history from middle-age than from older patients [2]. A recent study [10] revealed physicians have age bias when examining and treating sexual dysfunctions, as they were more likely to attribute sexual dysfunction in older age to physical issues and recommend pharmaceutical treatment.

Medical treatments offered specifically to older adults to assist in engaging in sexual practices reflect the medicalization of sexual function in later life. PDE5 inhibitors for treating erectile dysfunction (ED) [30] and the “pinking” Viagra [15] demonstrate the intersection of the medicalization of ageing with sexuality. Medicalization of older adults’ sexuality devalues nonpenetrative sexual practices by offering medical solutions to enable performance, and pressures older adults to use these products to emulate youthful, “normative” sexuality [21, 24]. The movement toward the medicalization of sexuality has led to a new norm in which engaging in sexual activity and preserving sexual function are criteria for successful ageing [20]. Gott [12] questioned whether this newly constructed need for sexual fulfillment in older age is more prescriptive than liberating. On one hand, it normalizes the desire for sexual activity later in life, yet on the other, it implies that successful or normative sexual activity is equated with vaginally penetrative intercourse and creates a binary perception of functional versus dysfunctional; thereby, restricting the meaning and range of sexual expression [17].

Studies report that men affected by sexual dysfunction also need to confront the socially-constructed nature of a sexual identity that is centered on penetrative sex [11]. The biomedicalization of sex can be a disappointing experience for some older adults, who feel there is too much emphasis on sexual function rather than on a partnered relationship and that they are captives of western cultural expectations of penetrative sex as the ultimate outcome of sexual desire [11]. Moreover, some older adults noted that the use of pharmaceuticals for erectile enhancement was ineffective, unsuitable from a relationship perspective or simply unpleasant [11]. Finally, the medicalization of sexuality in older age might disregard other important aspects of old age that can be important to one’s sexual function and satisfaction, such as altered body image, work, financial or family-related stresses or relationship issues [22].

Negative perceptions about later life sexuality may influence physicians’ medical approach [2, 10] and be internalized by older adults, thereby inhibiting or interfering with healthcare seeking [3, 13] or levels of sexual activity and interest [16]. It is therefore imperative to further assess the beliefs and attitudes that contribute to physicians’ perceptions and responses. The present study used a qualitative approach to examine how physicians evaluate sexual function in later life and how they treat sexual dysfunction in older vs. younger patients.

## Methods

### Sample and procedure

The sample consisted of 38 physicians practicing in Israel. The study was funded by The Israel National Institute For Health Policy Research and approved by the Helsinki committee of Meir Medical Hospital and the Institutional review board of the social work school at Bar Ilan University Institutional Review Board. Inclusion criteria for the study were specialization in gynecology, urology or family medicine, or certification in human sexuality by the Israel Society of Sex Therapy (ISST). Most participants (*N* = 23) were identified through personal contacts of the researchers and specialized in family medicine, urology or gynecology. An additional 15 participants were certified as sexologists by the ISST, the European Society of Sexual Medicine, the European Federation of Sexology or the American Association of Sex Educators, Counselors and Therapists, in addition to their medical specialty. These physicians were identified using the list of certified sexologists that appears on the ISST website [19]. They were approached by email or phone to be interviewed for the study. No incentives were offered. Efforts were made to include equal representation of genders and specialties. Participation was not limited by age. The characteristics of the physicians who participated in the study are presented in Table 1.

**Table 1:** Demographic characteristics of participants (*N* = 38)

Characteristics	*N/M*	%
Age, years	*M* = 49.84, *SD* = 10.18	
Seniority, years	*M* = 20.37, *SD* = 10.20	
Country of origin		
Israel	30	78.95
United States	1	2.63
South America	1	2.63
Europe	3	7.89
Eastern Europe	2	5.26
Russia	1	2.63
Sex		
Male	22	57.89
Female	16	42.10
Medical specialty		
Family Medicine	17	44.74
Urology	6	15.79
Gynecology	11	28.95
Rehabilitation	1	2.63
Psychiatry	3	7.89
Training in Sexology		
Yes	15	39.47
No	23	60.53

### Data collection

To explore participants’ perceptions of later-life sexuality, data were collected through in-depth, personal interviews at a time and place of their choosing (most often at the physician’s workplace). Research objectives and a systematic review of the literature (authors’ own) were used to design the interview guide, which deliberately covered broad topics, with the goal of revealing physicians’ perspectives on older adults’ sexuality. The interview guide is presented in Table 2.

**Table 2:** Interview questions posed to family physicians for the qualitative analysis

1	How do you define sexuality?
2	Tell me about sexual function in old age.
3	Do the reasons for engaging in intimate relations differ between young adults and older adults?
4	In your opinion, what reasons might elderly individuals have for refraining from engaging in intimate relations?
5	In your opinion, which factors might influence the levels of sexual function and sexual satisfaction in old age?
6	How do you think society/the media perceives sexuality among the elderly?
7	How does treatment of sexual function differ between younger adults and older adults?
8	Tell me about contacting/referring patients to different specialists in relation to sexual function difficulties among the elderly.
9	What are the advantages/disadvantages of contacting different specialists in relation to sexual dysfunction in old age?
10	In your opinion, how is it possible to create open lines of communication about sexual function between elderly patients and their physicians?

Prior to the interview, participants were given a general statement about the rationale and aims of the study. Consent was obtained verbally by each physician prior to the interview. Confidentiality and anonymity regarding the names of participants and their practices were assured. Interviews began in December 2016 and were completed by April 2017. Five interviewers with a social science background (three had additional training in sex therapy) conducted the interviews.. The interviewer were trained by two of the authors (AGM and LA) in two stages: first, interviewers read the interview guide with the trainers and were directed on how to ask the questions and conduct the interview. In the second stage, the trainers listened to the interviewers and provided feedback. Each interview lasted about 45 min to 1 hour. They occurred in the interviewees preferred location.

### Data analysis

After completion, all interview were transcribed verbatim and the data were analyzed thematically. Initially, a line-by-line, open-coding analysis was employed [29]. Analysis did not use preconceived codes, but allowed themes to emerge directly from the text (J. W [5].). The researcher (AGM) first read each interview transcript line by line, jotting down notes to capture and identify initial units of meaning (categories) emerging from the data. Next, codes were grouped into main themes to identify variations in responses. Two researchers (LA and IL) then reviewed the larger themes and discussed them with AGM. In a second reading of the transcripts, the researchers gradually detected associations between themes and sub-themes related to context and content. They compared all completed interviews so as to consolidate meaning and agree on a theoretical construct [28]. Finally, the core themes or main categories that emerged from the data were reordered conceptually and placed back into context, making it possible to analyze and integrate large amounts of data and generate abstractions and interpretations [27].

### Sources of trustworthiness

The trustworthiness of the analysis was enhanced by using different interviewers. A larger number of reviewers serves as an i*nvestigator triangulation*, which is defined as the use of different observers or interviewers to balance out the subjective influences of individuals [9]. Trustworthiness was also enhanced by sharing and discussing the coding among the authors. The themes were discussed with four of the physicians who participated in the study, to obtain feedback. Several measures were taken to ensure the quality of the findings. The coding scheme presented in this paper was created following the analysis of about two-thirds of the interviews. This newly developed coding scheme was checked against the analysis of the remaining interviews. We also kept an audit trail, documenting all stages of analysis [6]. Finally, we provide a detailed description, which includes direct quotes from the text, to allow the reader to judge the proposed coding scheme [26].

## Results

Perceptions on older adult’s sexuality that emerged from the interviews were organized into three themes: What, why and how. *What*, referred to physician’s definition to what role sexuality plays in later life and what is considered *sex* and what are the differences between sexuality among younger vs. older adults. *Why*, referred to the reasons why physicians assume older adults experience sexual difficulties, and how these assumptions effect the diagnostic process. *How,* referred to how sexual difficulties were treated by physicians. Below is a detailed description of the main themes identified, based on direct quotes from the interviews.

### What (is the role of sex in later life)?

*What* referred to physician’s definition to what role sexuality plays in later life and what is considered *sex*? Physicians also described how sex differs between younger vs. older adults. Most physicians interviewed described sexuality in later life as an important and integral component of older adults’ wellbeing and quality of life. Physicians viewed sexual activity at older ages as “normal” and were willing to assist and discuss sexual dysfunction with their older patients. Many physicians, especially those with previous education in human sexuality or training in sex therapy, viewed enabling patients to express sexuality as an important part of their job, no different from any other aspect in which they offer healthcare:

*“A person is never too old or too sick to be sexually active. Our job as physicians is to enable people to be sexually active until the day of their funeral.”* (Matthew, Psychiatrist, Sexologist).

However, many physicians differentiated between young and old when they described and defined sexuality in later life. Some assumed older adults are less interested in engaging in sex and have less energy and as a result, have a lower frequency of sexual activity than younger adults do. In addition, some physicians assumed older adults are more tolerant and more accepting of a decline in sexual function than younger adults are:

*“I think the frequency (of sex) decreases. It's hard to say, because people don't really report to me about the frequency, and I don't really know what happens in people's bedrooms … But I feel there is a serious decline with age, across the years … ”* (Lily, Family Medicine).

In addition, when discussing sexuality in later life, physicians have specific assumptions about what older men and women want. According to many physicians, older men define themselves by their ability to achieve an erection and intercourse. In contrast, older women have a greater need for intimacy and emotional closeness. Based on these assumptions, both female and male physicians defined a successful sexual engagement in older age as the ability to achieve an erection and the ability to engage in penetrative sex:

*“For men, what is important is their sexual function, full penetration … women are not as interested in intercourse because of the pain due to vaginal dryness; however, they seek touch and relationship, so I prescribe lubricant or vaginal creams”* (Ruth, Family Medicine).

This approach, which assumes older adults definitely desire sex that includes penetration, also assumes that older adults are heterosexual. A few physicians admitted to assuming older patients are heterosexual. While they carefully address the issue of sexual orientation with young adults (e.g. ask if they are in a relationship and not imply that they have a boyfriend/girlfriend), they automatically assume older adults are in a heterosexual relationship:

*“You know, when we talk about sexual function of older people, I am still held captive by the idea that sexual function, that is more ah … heterosexual. I mean, I don’t recall asking older people about other types of relationships. It is like in older age, that goes without saying, but with younger adults, I will be much more careful and ask, “Are you in a relationship?” and not, “Do you have a girlfriend … ?” (Emma, Family physician)*.

However, some physicians, mostly those with training in human sexuality, differed in this matter. According to them, sex in older age is not only about penetration, but encompasses intimacy, warmth and touch. They emphasized that older adults engage in sex not just for penetration, but also to strengthen their relationship, feel closer to their partner, feel loved, and feel young again. They emphasized the importance of normalizing and calming the patient. These responses might be the message older adults were looking for and can explain why some physicians emphasize that sexuality involves much more than penetration and encourage their older patients to be creative in their sexual expression:

*“Sex is not a race and does not equal penetration. It is possible that older people would want intimacy without intercourse. Everyone can have it their way … just hugging, cuddling, stroking each-other”* (Joseph, Gynecologist and Sexologist).

According to this view, physicians cannot define sex, what enjoyable sex is, or what is the goal of sex. Physicians noted pleasure and orgasm can be obtained by many means beside penetration and when they asked their patients what they wanted, they were surprised to hear that older adults were sometimes satisfied with sex that did not include intercourse, but just hugging, kissing, caressing or giving/receiving oral sex:

*“When you ask people what is sex, they will say sex is a penis penetrating a vagina. But clearly, that is only one type of sex. Even if a woman has a decrease in her libido, or a man has decreased erectile function, they can still have wonderful sex if we help them define what sex is, and what the meaning of having sex is. Mutual pleasure could be obtained in many ways”* (Daniel, Rehabilitation Physician and Sexologist).

### Why (do older adults experience sexual problems)?

*Why* referred to the reasons why physicians assume older adults experience sexual concerns. Physicians viewed the reasons for sexual dysfunctions differently among older vs. younger adults. Most physicians assumed there was an organic problem. However, sexual dysfunctions among younger patients, were discussed in relation to psychological issues or anxiety:

*“With younger adults, I expect to see sexual dysfunctions that are more psychological, whereas with older adults I assume the sexual dysfunctions are more of a mechanical dysfunction, and not performance anxiety or other psychological disturbances … ”* (Don, Urologist).

Based on these working assumptions, when referring to older adults, physicians focused on the physical aspects of sexual function among older women (vaginal dryness, decreased libido due to hormonal changes) and men (erectile dysfunction and delayed ejaculation). When examining older patients, physicians tended to ignore psychological and emotional aspects, and first sought an organic source for the dysfunction.

Some sexologist physicians have noted other factors that may affect sexual dysfunction in later life, such as retirement, taking care of older parents or grandchildren, or the challenge of being alone again with their partner (empty nest syndrome). In fact, some physicians describe changes in later life as having an almost inevitable impact on sexuality. People who have completed early tasks of life, such as raising children and working, find themselves choosing how they want to spend their time. This according to some physicians, can lead to a very successful sense of new self-fulfillment and quality time for the relationship and intimacy, or could cause couples to drift apart and reveal gaps that were denied or repressed for years:

*“There are social changes … children leave the house, and you stay only with your partner. Sometimes intimacy grows stronger, but sometimes the opposite happens, and sex is like an explosive material … Also, often, when there is more free time, people go back and fight over things that happened or didn’t happen in the past”* (David, Gynecologist and Sexologist).

However, although sexologists have described a broader examination which included social, dyadic and psychological aspects, most physicians in the study assumed the source of the sexual dysfunction in older age was organic, and this assumption perhaps affected and biased the interventions offered. These assumptions effect the diagnosis process among young vs. older patients. For example, some physicians disclosed that while conducting regular procedures, they always notify the patient about possible effects on sexual function (if relevant); with older patients, they sometimes forget to do so, and it just *slips their mind*:

*“There are some ages, when I … for example when an 80-year-old man comes with a problem of hydrocele, water in his testicles, I sometimes 'sin' and do not ask about sexual function … and then they ask. It comes from them … ”* (Rene, Urologist).

### How (sexual concerns in later life are treated)?

*How* referred to how sexual difficulties presented by older patients were treated by physicians and what was their approach for intervention. According to physicians, solutions offered to older patients are more medication-oriented than those offered to younger patients. This indicates that older adults are treated with a biomedical approach. Medication was recommended for older adults more easily and rapidly than it was to younger adults. Physicians admit prescribing medications (e.g. oral medication, local creams or hormones) to older patients more quickly than they would prescribe them to younger patients. In addition, older adults are more likely to be referred to a urologist, whereas younger adults would be referred for therapy or counseling:

*“*W*ith older adults, I start much faster towards injections, because I don't trust the efficacy of testosterone, Viagra, Cialis, etc. I refer young adults to a sexologist, but I never do that with older adults, because the basic assumption is that the dysfunction is mechanical”* (Don, Urologist).

With younger patients, physicians described taking more time to understand whether the source of the dysfunction is emotional rather than functional. They try to avoid medication and provide more guidance in psychological issues relating to the sexual dysfunction. Physicians said that they discuss the importance of receiving relationship counselling and developing open communication with one’s partner, more than they would with older adults:

*“With younger adults, therapy is more psychological. Meaning, we will work more on couple therapy, sex therapy, emotional aspects of sexuality, how to focus during sex, and sexual techniques, we will work more on the emotional aspects … With older adults, we will focus on the organic and physical aspects, which we can treat with medication … ”* (Michelle, Gynecologist and Sexologist).

As a result of the different treatment offered to younger vs. older patients, some of the physicians reported differences regarding the involvement of the partner in the treatment. With older patients, to whom they tend to prescribe medication, they do not necessarily invite the partner. However, when they provide consultation or refer the patient to sex therapy (most likely the younger patient), they emphasize the importance of the partner’s participation in the process:

*“With young adults, I try to give more guidance around the relationship, and do not rush to prescribe medication. I talk with them about the importance of counseling and sharing the difficulties with their partner. I will want their partner to come and will explain to both of them that their difficulty is not physical, but is based on their experiences, low self-esteem or anxiety.” (Emma, Family Medicine)*.

This biomedical approach seemed to intersect with and build upon the assumption that older adults want penetrative sex, and that the etiology is attributed to dysfunction that occurs at older ages. Physicians perceive that they need to provide treatment that will enable penetrative sex. Penetration was perceived as the ultimate successful result of their intervention. Therefore, older men were offered PDE5 inhibitors to enable them to achieve an erection, and lubricants or estrogenic creams were offered to older women, so they will not experience pain during intercourse. Physicians described how they plan to assist older patients to achieve an erection, starting with screening their hormonal levels, prescribing oral pharmacotherapy (PDE5) and offering intracavernosal self-injection therapy, vacuum pump devices and even penile implants.

However, some physicians, mostly those with education in human sexuality or training in sex therapy expressed more egalitarian statements regarding the therapy they provide to older adults. According to them, there is no real difference in how they treat older vs. younger people because sex therapy is a psychological treatment centered around peoples’ relationships with their partners. Therefore, they examine patients regardless of their age, and many times offer similar treatment plans to younger and older adult:

*“I treat people, a woman, whenever and however she is. When I take medical history, I am interested in how she defines the problem, and I do the same for a young 25-year-old, a 42-year-old woman, or a 72-year-old lady. It is all the same to me”* (Neomi, Gynecologist and Sexologist).

The physicians who had these perceptions on sexuality emphasized the importance of normalizing and calming the patient as part of their intervention. According to them, when physicians rush to offer medication, they validate that something is wrong with their patient’s sex life that needs to be fixed. However, even some sexologists were only able to adopt broader definitions of sex after trying conventional treatments (such as Viagra or lubricants) that failed. Only then, they offered their older patients a different perspective on sex:

*“I had a patient (60) who had painful intercourse and I wasn't sure I could help her anymore, so the next stage was helping her, and her partner adapt to the idea of sex that does not include intercourse and not to view penetration as a sacred goal … ”* (Mellie, Gynecologist).

## Discussion

The present study investigated physicians’ perceptions of later-life sexuality and how it differs from their approach to young adults. This study is important, as physicians’ attitudes toward sexuality at older ages has been a neglected area of study, despite their relevance to the quality of life and well-being of older adults. In addition, this study raises questions regarding equality and justice in health services supplied to the general population and to older adults, in particular.

Consistent with previous research [2, 10], findings in the current study indicate that some physicians still have negative attitude towards later life sexuality. However, this study also reflects a movement and change towards a promising and more positive approach to older adult’s sexuality. Most of the physicians expressed the importance of sexuality across all ages and some have also described using biopsychosocial approach for treating sexual dysfunctions in later life. While this change is noticeable and appreciated, to the majority of physicians focused on the biological, rather than psychological, social or cultural aspects when discussing later life sexuality. Older patients’ sexual concerns were treated from a biomedical perspective, while those of younger adults were considered from a broader perspective that included biological, psychological and social dimensions. Misconceptions and stereotypes about later life and older adults can interfere with healthcare seeking, as well as with diagnostic and treatment recommendations [3, 10].

Even though the influences of medicalization and biomedicalization on sexuality and sex therapy have increased in recent decades [31], the current study demonstrated the intersection between the medicalization of sex and the ageing process [30]; indicating the presence of ageism and inequality in the treatment provided. Older adults are less likely to be referred to sex therapy, based on the assumption that declines in sexual performance than are natural at this age [10]. Although sexual dysfunction can be triggered by many psychological and social variables, such as performance anxiety, poor body image, changed status at work or in society, rigid sexual beliefs and myths, or other stressful factors (e.g. financial, work [22];), most physicians dismissed these issues. An integrated approach combining oral medication and psychotherapy has been shown to have a superior therapeutic outcome compared to pharmacotherapy or psychotherapy alone [22]. Yet, according to the interviewees, prescription of oral medication for older adults is usually not accompanied by a referral to psychotherapy.

Offering oral medication dismisses many psychological factors possibly associated with sexual dysfunction in later life, and signifies that physicians are ensnared in common social norms that equate sex with intercourse. Although age-related changes and sexual health in middle-aged and older adults are evident and erectile dysfunction appears to be more common among older men [1, 4], many older adults seem to be satisfied with various forms of sexual engagement other than vaginal penetration (e.g. physical contact such as hugging, cuddling, oral sex, masturbation or clitoral stimulation [11, 18]. However, the biomedicalization of sex, western cultural expectations and heterosexual cultural norms have influenced society to perceive penetrative sex as the ultimate outcome of sexual desire [11, 31]. In the current study, physicians described their treatment plans as focused on the ability to achieve penetrative sex rather than on exploring options more compatible with how their older patients define sexual satisfaction and how they can achieve it.

The treatment offered to older adults based on physicians’ perceptions could imply that sexual activity among older adults is defined solely by penetrative intercourse and that participation in such activity is requisite for successful ageing, particularly for older men [17]. However, several physicians held broader and more inclusive views around what is defined as sex; thus challenging and rejecting hierarchical and heteronormative understandings that penetrative intercourse constitutes *real sex* [8]*.* According to McCarthy, Farr, and McDonald [25] focusing on sharing pleasure as a couple is the key for mutual connection and sexual satisfaction, while focusing on sexual performance, intercourse and orgasm may lead older couples to frustration, embarrassment, or avoidance.

The current study did not attempt to represent the general population of physicians; rather, it presents perceptions and views on late life sexuality and the interactions between physicians and older patients regarding these issues. The study provides important insights about the way sexuality in later life is perceived and treated by physicians. Although most physicians expressed its importance, they tended to focus on the medical aspects and work under the perception that sexual dysfunctions among older adults are biological in origin. Sexual dysfunctions in later life were not addressed from an integrated, holistic perspective that included psychological, social and behavioral aspects, which might have created or contributed to the dysfunction. Regarding treatment methodology, most physicians demonstrated a medically-oriented approach, offering medications to enable penetrative sex, reinforcing heteronormative constructions, limiting sexual expression to penile-vaginal penetration, and included intercourse as a criterion for successful ageing [21].

### Limitations

Although this study provides important insights on how physicians perceive older adult’s sexuality, several limitations should be considered. First, qualitative research does not allow inclusion of large populations. In addition, physicians were asked to reflect on previous experiences and treatments of patients rather than on real-time events. Finally, we did not ask questions with regard to age groups, even though there are differences in the sexual expression and function between age subgroups in later life.

### Implications

The health care system can be improved by acknowledging that problems older adults present are occasionally viewed, diagnosed and treated differently than are those of younger adults. This does not mean all assessments, treatment procedures, and processes should be standardized across age, but it does mean providing equal and adequate care to all patients. It means creating care that respects the unique needs of the individual, regardless of his or her age. On the individual level, physicians need to explore their own stereotypes on sexuality in later life and how the age of their patient affects their decision-making. If they offer sex counseling to younger people, they should consider doing the same for older people. This is not to standardize treatment, but to improve effectiveness. Improved education and training in both human sexuality and aging are vital to reducing age bias among those treating sexual problems in later life.

Health plan leaders should consider protocols for offering medication for sexual dysfunctions (e.g., Viagra™) in order to minimize age bias. This protocol should consider both partners if possible, a recommendation for couple/sex therapy, intake on the etiology of the problem and the desired goal of the patient and his or her partner, and a follow-up meeting. Finally, on a governmental level, sexual education programs developed by the health ministry should be modified for different age groups. Most sexual education programs focus on youth and young adults; yet, with the increased life expectancy and number of active, older adults, sexual education programs that are modified for later life need to be developed. Currently, education on later-life sexuality is conveyed by the pharmaceutical community, focusing on medications rather than intimacy, touch and communication. This type of sexual education can shape public opinion, policies and quality of treatment given to older adults in relation to sexual difficulties. Continuing education for physicians should address sexuality from a broader perspective, which includes much more than sexual-intercourse between partners of the opposite sex. Sexual difficulties should also be viewed not only as part of age-related physiological changes, but should also consider various other changes in older people’s lives. Hopefully, this type of approach will affect the type of intervention used and prevent physicians from viewing older adult’s sexual difficulties as a medical condition or as a problem that needs “fixing”.

## Conclusions

The findings highlight the potential for differential treatment of older adults, which is based on age, rather than on other objective reasons. We suggest that physicians should apply a biopsychosocial model when treating sexual dysfunction among older adults, in the same way they tend to do when treating younger adults. In addition, it is important to inform older adult patients that there is no correct or clearly defined way to approach sexuality in later life. In addition, the findings also suggest examining sexual function at older ages from a dyadic perspective, understanding how the relationship affects sexual function and is affected by it. However, although physicians described that they ask about the relationship and about the partner’s opinion, in most cases they did not include the partner when developing a treatment plan. We recommend involving the partner in all aspects of treatment planning. In addition, based on the importance of understanding the sexual dysfunction in terms of the dyadic unit, we suggest physicians consider recommending psychotherapy (e.g. counseling, sex therapy) in addition to or instead of oral medication, as they often do with younger adults.

## Data Availability

The data sets generated during and/or analyzed during the current study are not publicly available due to confidentiality.
